# Characteristics of Patients Who Complete Suicide and Suicide Attempts While Undergoing Treatment in Norway: Findings from Compensation Claims Records

**DOI:** 10.3390/ijerph20054083

**Published:** 2023-02-24

**Authors:** Sanja Krvavac, Martin Bystad, Rolf Wynn, Ida Rashida Khan Bukholm, Billy Jansson

**Affiliations:** 1 Department of Psychiatry, Helgeland Hospital Trust, 8802 Sandnessjøen, Norway; 2 Department of Health and Care Sciences, UiT The Arctic University of Tromsø, 9037 Tromsø, Norway; 3 Division of Substance Use and Mental Health, University Hospital of North Norway, 9019 Tromsø, Norway; 4 Department of Clinical Medicine, UiT The Arctic University of Tromsø, 9038 Tromsø, Norway; 5 Department of Education, ICT and Learning, Østfold University College, 1757 Halden, Norway; 6 The Norwegian System of Patient Injury Compensation, 0130 Oslo, Norway; 7 Faculty of Landscape and Society, The Norwegian University of Life Sciences, 1430 Ås, Norway; 8 Department of Psychology and Social Work, Mid Sweden University, 831 25 Ostersund, Sweden

**Keywords:** diagnosis, medical error, suicide attempters, suicide completers, suicidal risk

## Abstract

The aim of this study was to identify characteristics that differentiate patients who complete suicide (SC) from patients with suicide attempts (SA) while undergoing treatment in Norway. We examined data from the Norwegian System of Patient Injury Compensation (Norsk Pasientskade Erstatning—NPE). Data were extracted from NPE case records from a 10-year period (2009–2019) for 356 individuals who attempted (*n* = 78) or died by (*n* = 278) suicide. The two groups differed significantly in the types of medical errors identified by experts. Inadequate suicide risk assessment tended to be proportionally and significantly more prevalent among SC compared to SA. There was a weak but significant trend that SA had received medication only, whereas SC had received both medication and psychotherapy. There were no significant differences with respect to age group, gender, diagnostic category, number of previous suicide attempts, inpatient/outpatient status, or category of responsible clinic. We conclude that suicide attempters and suicide completers differed in terms of identified medical errors. Focusing on the prevention of these and other types of errors could help to reduce the number of suicides of patients in treatment.

## 1. Introduction

Suicidal behavior culminates in approximately 800,000 deaths per year globally as the second leading cause of death among 15–29 year-olds [1]. A major task for psychiatric inpatient units is to treat patients with a high risk of suicide. In Norway, more than 50% of admissions to acute psychiatric units are due to patients’ increased suicide risk [2], and a considerable proportion of these patients are involuntarily admitted [3,4].

In psychiatric institutions, it is not uncommon that patient safety is at risk due to medical errors [5]. One study found that one in five patients in psychiatric settings has experienced a medical error or adverse event, and 56.6% of all events were conceptualized as preventable [6]. In some instances, medical errors may be a contributing factor in the occurrence of suicide attempts or completed suicides and it is important to identify and prevent such medical errors.

Several studies have compared Suicide Attempters (SA) and Suicide Completers (SC), which are two coinciding but at the same time distinctive populations [7,8,9,10]. More than 90% of SA do not die by suicide; however, 60% of SC die in their first attempt [11]. Compared to the SA, the SC are more likely to be males over 45 years of age [12], live alone [9] or experience work-related stress and financial problems [7].

Suicide methods may also vary according to subgrouping within SA or SC. SC often use highly lethal methods such as shooting or hanging, whereas about 70% of SA use less lethal methods, such as cutting and overdosing [13,14]. In addition, whereas SC typically had prior contact with general practitioners (GP) and were less likely to have had a prior history of suicide attempts [12], SA typically had a history of previous SA and prior psychiatric treatment [8].

Clinical predictors, such as depression, represent a significant risk factor both for SA and SC [7,9,15]. However, SA are more likely than SC to be diagnosed with comorbid anxiety disorder and tend to be more socially isolated [16]. Furthermore, bipolar disorder, depression, severe alcohol misuse, and suicide ideation are more frequent in SC compared to SA [13,17]

Several longitudinal studies report a higher risk of SC for psychiatric inpatients, compared to a primary healthcare population [18,19]. Research shows that suicides are more likely to occur during inpatient psychiatric hospitalization [20,21] or during the first few weeks after discharge [21,22]. Moreover, inpatient population predictors of SC comprise a diagnosis of personality disorder (particularly borderline personality disorder), substance abuse, and a previous history of suicide attempts [19].

Some previously described medical errors that may increase the risk of suicide during hospitalization are (1) an incomplete assessment of suicide risk or an insufficient follow up after suicide risk assessments, (2) that the patient has access to dangerous objects or drugs, (3) a lack of sufficient or qualified healthcare professionals [15,23].

In a prior study [24], we examined the characteristics of SC in detail, drawing on data from Norwegian compensation claims. In the current study, we build on and expand our scope by comparing characteristics of patients who attempted suicide to those who completed suicide, seeking to gain more understanding about the similarities and differences between the two groups and the types of medical errors that have been identified by experts. This knowledge may hopefully be used to improve the work relating to the prevention of suicide attempts and completed suicides of patients undergoing treatment.

## 2. Materials and Methods

### 2.1. Measures

Diagnostic categories. ICD-10 [25] F-diagnoses were drawn from the case records. We categorised the diagnoses as follows: (1) F20–F29 Schizophrenia and psychotic disorders, (2) F30–F39 Mood disorders, (3) Other diagnoses, including personality disorders, substance use, eating disorders, anxiety disorders.

Patient status. A distinction was made between patients who had been hospitalised (or on leave from hospital) when the suicide attempt or suicide occurred and patients who were outpatients at that time.

Health care type. The types of health care institution data were extracted from patients’ clinical case records and coded as a dichotomous variable: (1) psychiatric institutions where participants were treated and/or hospitalised and (2) other institutions (public general hospitals, community health care services, private practice health care specialists, general practitioners’ clinics, addiction treatment services, and private hospitals working for the public health services; this category also includes somatic units).

Treatment received. The treatment received was categorised according to complexity. This information was drawn from the patients’ electronic health records and the Norsk Pasientskade Erstatning (NPE) expert evaluations. The categories were: (1) Medication, (2) medication and psychotherapy, and (3) electroconvulsive therapy (ECT), medication, and psychotherapy.

Medical errors. This measure was extracted from NPE expert evaluations. The experts were tasked to assess whether the institutions and health professionals had treated the patients according to best practice and an acceptable level of care or whether any error had been committed in the patients’ care and treatment. In the present study, the medical errors that had been identified were coded as: (1) Insufficient level of observation of symptoms and lack of safety measures (i.e., a failure in observing all the patient’s symptoms); (2) inadequate suicide risk assessment (i.e., a lack of staff qualifications and/or experience); (3) insufficient/delayed diagnostic assessment (i.e., wrong and/or delayed assessment); (4) inadequate treatment (i.e., errors in medication and/or insufficient follow up). For instance, if the NPE expert had found that the patient’s suicide could have occurred because the patient was allowed to exit the psychiatric ward alone despite being assessed as being at a high risk of suicide, this would be classified as a medical error (insufficient follow up, type 4 described above).

### 2.2. Procedures and Participants

The study was based on data from the NPE [26], drawing on information from expert examinations and psychiatric case records from 2009 to 2019. The study included altogether 356 individuals who attempted suicide (SA) (*n* = 78) or completed suicide (SC) (*n* = 278) in Norway [25].

A central role for the NPE is to process compensation claims from people who think that either themselves or someone in their family had been injured as a consequence of being treated at a Norwegian health institution. Both private and public institutions are covered by the NPE. The role of the NPE is to evaluate whether there has been a failure in the treatment given, and whether the failure was the reason for the damage the patient experienced.

Almost half of the participants received approval of the compensation claims (49.0%, *n* = 175). A slight majority were male (55.9%, *n* = 199) and belonged to the age category 30 to 59 years (52.8%, *n* = 188). Three-quarters received treatment at psychiatric units (75.8%, *n* = 270). Most had received a diagnosis of mood disorders (55.9%, *n* = 199) or schizophrenia/psychotic disorders (17.1%, *n* = 61). The remaining patients (27.0%, *n* = 96) had received a number of different diagnoses, i.e., different personality disorders, learning disabilities, dementias, substance use disorders, different anxiety disorders, OCD, and PTSD. As there were few patients in each diagnostic group, we combined these into one ‘other’ diagnostic category for the purpose of statistical analyses.

More than half had an inpatient status (*n* = 204, 57.3%). SC constituted the majority of the sample (78.1%, *n* = 278). The following criteria were set for study inclusion: (1) Death by suicide or attempted suicide; (2) The participant was receiving treatment when the suicide attempt or completed suicide occurred; (3) A compensation claim had been filed by the next-of-kin or the participant at the website of the NPE (www.npe.no). Those who had attempted suicide mostly applied themselves, whereas next-of-kin applied on the behalf of those who had completed suicide. The study followed the Declaration of Helsinki and had received approval by the Head of the NPE and the NPE Research Assessment Committee (Personvernombudet).

### 2.3. Interrater-Reliability Analysis

A clinician (H.B.) performed interrater reliability analyses (IRR) of types of medical errors, treatment types, outpatient/inpatient status, and medical errors. Thirty random cases for the inpatient vs. outpatient variable were extracted, resulting in an overall agreement of 86% (Cohen’s Kappa = 0.86, *p* ˂ 0.001). Sixty random cases were extracted for both treatment types and medical errors, with an overall agreement of 70% obtained for treatment types (Cohen’s Kappa = 0.72, *p* ˂ 0.001), and 90% for medical errors (Cohen’s Kappa = 0.99, *p* ˂ 0.001).

### 2.4. Data Analytic Procedures

Chi-square tests (likelihood ratios) were used to examine the interrelation between the categorical variables and the dependent variable (SC vs. SA). The odds ratio (OR) was used as an effects-size statistical measure

In a next step, using a joint multinomial sampling scheme, we calculated Bayes factors for contingency tables [27,28], with the purpose of determining the absence or presence of effects. By applying the method of Bayesian hypothesis testing, we are able to examine the relative degree of evidence against or for the alternative hypothesis [29]. The Bayes factor (BF) is a numerical value that quantifies to what degree a hypothesis can predict the empirical data in comparison to a competing hypothesis. The likelihood of the data given the alternative hypothesis relative to the likelihood of the data given the null hypothesis is expressed by BF_10_. As an example, a BF_10_ of 6 suggests that the empirical data are 6 times more likely if H₁ were true than if H₀ were true. If BF_10_ is 1, this will imply that the data are equally likely to occur under both hypotheses. A BF_10_ under 1 should be taken in favor of H₀, as a BF_10_ = 0.25 will suggest that the empirical data are 4 times more likely if H₀ were true than if H₁ were true (which may be written: s BF_01_ = 4 [BF_10_’s inverse BF_01_; = 1/BF_10_]). Drawing on the classification scheme of Jeffreys (1961) [30], we classify Bayes factors between 1 and 3 as *anecdotal evidence*, Bayes factors between 3 and 10 as *moderate evidence*, and Bayes factors above 10 as *strong evidence*. We performed the analyses with the help of the software JASP [31].

## 3. Results

### 3.1. Descriptive Results

Frequencies for all measures are presented in Table 1, Table 2 and Table 3. Results showed that the majority of the SA and SC were in the 30–59 years category. A majority of the SC were males, and nearly half of the SA were males. A majority of the SA and SC were diagnosed with mood disorders. The major part of SA and SC were inpatients at psychiatric units, and most received either medication only or medication in combination with psychotherapy. The most common medical error with the SA was an insufficient level of observation and lack of safety measures, whereas for the SC, the most common error was an inadequate suicide risk assessment.

### 3.2. Tests of Independence

**Figure 1:**
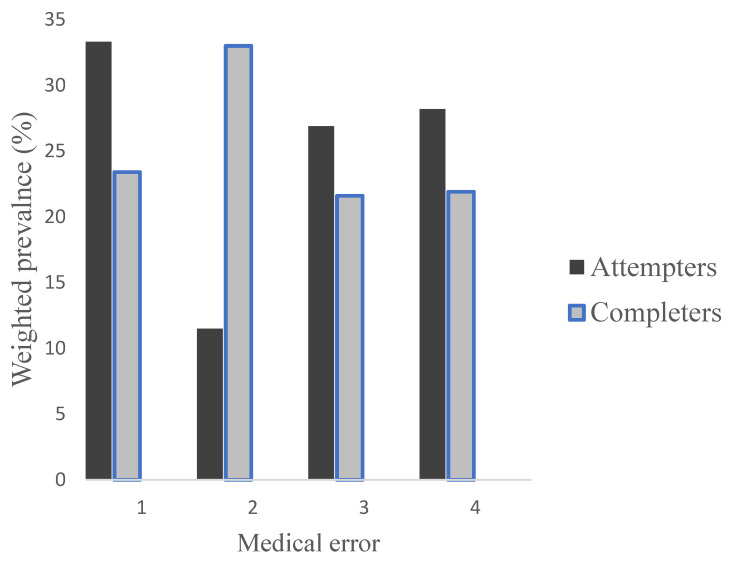
Weighted frequencies (% within each group) for each medical error category by group (attempters vs. completers). 1—Insufficient level of observation/lack of safety measures; 2—Inadequate suicide risk assessment; 3—Inadequate/delayed diagnostic assessment; 4—Inadequate treatment.

We did not find any evidence that the SA and SC differed with respect to age and gender (non-significant effects and BF of less than 1 were indicative of support for H₀, see Table 1), nor did we find any evidence that SA and SC differed with respect to diagnoses (non-significant effects and BF of less than 1 were indicative of support for H₀, see Table 2). Furthermore, we found no evidence (non-significant effects and BF of less than 1 were indicative of support for H₀) that SA and SC differed with regard to patient status or type of health care. However, SA and SC differed substantially with regard to medical errors. That is, apart from the significant *p*-value, the Bayes factor found evidence for H_1_ compared to H_0_, suggesting that the data are approximately 22 times more likely to occur under H_1_ compared to H_0_ (see Table 3)_._ As the chi-square test is an omnibus test, post-hoc tests were conducted to explore the relative contribution of cells to the chi-square analysis by calculating the adjusted residuals of each cell (equivalent to a z-score). Cells which have a standard residual larger than 1.96 are viewed as significant (for a = 0.05), but the z-scores were corrected for multiple comparisons (i.e., four contrast, *p* ≤ 0.0125). Three contrasts were associated with adjusted residuals corresponding to *p* ≤ 0.0125. As shown in Figure 1, inadequate suicide risk assessment tended to be proportionally more prevalent among SC compared to SA (adj. res = 3.7, *p* = 0.0001). Finally, even though proportionally more prevalent among SA compared to SC, differences related to insufficient level of observation/lack of safety measures (adj. res = 1.8, *p* = 0.073), inadequate/delayed diagnostic assessment (adj. res = 1, *p* = 0.32), and inadequate treatment (adj. res = 1.2, *p* = 0.23) did not reach statistical significance.

Finally, there was a weak but significant (*p* = 0.039) effect indicating that SA and SC differed on type of treatment, suggesting that whereas the SA group tended more often to have been treated with medication only compared to SC (adj. res = 2.2, *p* = 0.028), the SC group tended to be treated more often with a combination of medication and psychotherapy compared to SA (adj. res = 2.5, *p* = 0.012). However, the BF was less than 1, indicative of support for H₀, see Table 3.

## 4. Discussion

The main finding in our study was that the two groups differed in the type of observed medical errors, as judged by the experts. Most importantly, an inadequate suicide risk assessment tended to be proportionally more prevalent among the SC compared to the SA. Previous studies have found that the SC group may have had a higher suicidal ideation [14], to have been more unwilling to report suicidal ideation [7,32], have had more comorbid medical illness [13], and have had a higher tendency to consume alcohol or drugs prior to suicide [7,33].

However, these factors are relatively non-specific and difficult to rely on in a clinical setting to identify a heightened risk of suicide. Clinically, it may pose a challenge to identify patients with a high risk of suicide attempts. Although suicidal thoughts are common in patients, actual suicidal attempts are rarer [34]. Consequently, a more rigorous regime of suicide risk assessments using structured instruments could be called for.

SC tend to have higher Montgomery–Åsberg depression rating scale ratings and higher suicide assessment scale scores than SA [17]. However, even these instruments, specifically designed for the assessment of the short-term risk of suicide, have a level of specificity and sensitivity that may result in an unacceptable rate of false negative and/or false positive cases [15]. Unfortunately, there are currently no psychological tests, clinical assessments or biological markers that can with a high degree of sensitivity and specificity predict the short-term risk of SA or SC [15,35,36], which could be used to identify those of high risk in order to provide these patients with adequate psychiatric services.

It is a further challenge that many mental health professionals lack specific suicide risk assessment training and treatment practice [37,38]. A thorough suicide risk assessment requires that the clinician has sufficient knowledge about the patient’s individual circumstances and any risk factors. Examples of factors of importance in addition to the patient’s suicidal thoughts/plans may be the patient’s psychiatric history and prior suicide attempts, current diagnosis, effect of ongoing treatment (if any), any recent losses, substance misuse, serious physical illness, sources of emotional support, reasons to live, etc. Systematically training clinicians in clinical suicide risk assessment and in providing treatment to suicidal patients, in combination with the structured use of those instruments that are available, may nevertheless be the best option for reducing the occurrence of suicide attempts and completed suicides.

We do not know why the medical errors in our sample occurred. However, the four types of medical errors were related to insufficiencies in staff qualifications, experience, and/or behavior (i.e., errors in observation, assessment, follow-up). Securing qualifications and experience among staff may in part help to address these challenges. Moreover, emphasising sufficient staff levels will allow staff the necessary time to properly conduct the needed observations, assessments and follow-ups.

In our study, the two groups (SC and SA) did not differ statistically with regard to the type of health care they received (psychiatric institution or other part of the health service), but there might nevertheless be differences in the type of professionals that are responsible for the treatment of the two groups of patients; however, we lack more detailed data on the professionals involved.

We also found some weak but significant trends regarding treatment types. The results showed that the medication only tended to be proportionally more prevalent among SA compared to the combined treatment of medications and psychotherapy that was more common in the SC group. This could reflect differences in symptomatology between the two groups, i.e., that the SC group tended to be judged more in need of or more suitable for psychotherapy. However, even though frequentist statistics suggested the presence of a group difference, the Bayes factor suggested an absence of difference.

Further analyses suggested that the SA and the SC groups did not differ significantly with respect to age, gender, diagnoses, and the number of previous suicide attempts. Previous studies have suggested that SA, when compared to SC, are more typically women, socially isolated, with a history of psychiatric treatments, and a less aggressive method for the attempts [7,8]. Previous studies have also suggested that the SC are more typically older males, live alone, have a comorbid somatic illness, choose the more lethal methods, and are followed up mainly by primary health care providers [7,8,10,12].

The present study has some limitations that need to be addressed. The first limitation is that the sample consists of data from the NPE reported by patients themselves or by their family members. A significant proportion of patients in treatment who attempt suicide or complete suicide are, for various reasons, likely not to be registered with the NPE. Consequently, the results are based on a selected group of patients that may not be fully representative of all SA and SC patients in treatment in Norway. However, these selected patients may reveal important information regarding reoccurring medical errors and identify areas that should be addressed in order to improve Norwegian psychiatric services. Nevertheless, in order to investigate medical errors and risk factors for SA and SC, future studies should rely on a larger and less selected sample size.

Another limitation is that the identification of medical errors was based on expert evaluations. Although we believe this to be a thorough process, it will necessarily involve a degree of subjective judgement on the part of the expert. Our data were obtained from the records of the NPE. We had access to a limited number of anonymised variables. We were unable to analyse the methods used by the patients in the suicides and suicide attempts due to incomplete data, possibly because the methods were not consistently reported to the health care facilities and therefore not to the NPE.

Information on the patients’ ethnicities was not collected by the NPE due to current regulations. We believe that the historically homogenous nature of Norway’s population may underlie the lack of attention to ethnicity as a possible factor of importance in the health care services. As the number of people of different ethnicities increases in Norway, this factor is likely to become more important in research on medical errors.

A prospective clinical study including a number of predefined and validated measures would not only facilitate any comparison between studies, it would also help to enhance the competencies at the participating psychiatric units. In addition, longitudinal prospective studies are needed to exclude alternative explanations for the obtained results.

## 5. Conclusions

Suicide attempters and suicide completers differed significantly in the types of medical errors identified. Inadequate suicide risk assessment tended to be proportionally more prevalent among SC compared to SA. Regarding the type of treatment the two groups had been offered, there was a weak but statistically significant finding that the SA had received medication only, whereas the SC had received combined medication and psychotherapy. There were no significant differences with respect to age group, gender, diagnostic category, number of previous suicide attempts, inpatient/outpatient status, or category of responsible clinic.

In order to increase knowledge and clinical proficiency, the important topic of suicide assessment should be addressed at different levels of education and training of the health professionals who are responsible for such assessments. By drawing on a spiral-learning model [39], the level of complexity can be increased as the students/professionals gain more knowledge and training.

Systematically addressing errors in order to improve the treatment and care of patients with psychiatric illnesses could help to reduce the number of suicides of patients in treatment. A first step would be to inform clinicians and other stakeholders that our findings suggest that even more attention should be paid to conducting thorough suicide risk assessments of patients. In order to reach clinical staff, this information might be provided in a clinical environment, such as during in-house training, grand rounds, or similar meetings.

Although clinical suicide assessment training combined with the use of structured tools for assessment will not help to identify all cases, this is nevertheless the best approach for identifying those who have the highest risk of suicide and suicide attempts.

## Figures and Tables

**Table 1:** Sociodemographic factors of suicide attempters (SA) and suicide completers (SC).

Sample Characteristics	Suicide Attempters		Suicide Completers		
	N	%	N	%	Statistics
**Age groups**					*χ^2^*(2) = 2.08, *p* = 0.353 BF_10_ = 0.07
0–29	29	37.2	90	32.5	(Reference)
	Adj. res. = 0.8		Adj.res. = −0.8		
30–59	42	53.8	146	52.7	OR = 1.12 (0.65–1.92)
	Adj. res. = 0.2		Adj. res. = −0.2		
60–	7	9.0	41	14.8	OR = 1.89 (0.76–4.66)
	Adj. res. = −1.3		Adj. res. = 1.3		
**Gender**					*χ^2^*(1) = 2.08, *p* = 0.149 BF_10_ = 0.46
Male	38	48.7	161	57.9	(Reference)
	Adj. res. = −1.4		Adj. res. = 1.4		
Female	40	51.3	117	42.1	OR = 0.69 (0.42–1.14)
	Adj. res. = 1.4		Adj. res. = −1.4		

**Table 2:** Diagnoses of suicide attempters and suicide completers.

	Suicide Attempters (Reference)		Suicide Completers		
	N	%	N	%	Statistics
**Diagnosis ICD10**					*χ^2^*(1) = 0.84, *p* = 0.658 BF_10_ = 0.45
F20–F29 Schizophrenia/psychotic disorders	16	20.5	45	16.2	OR = 0.79 (0.37–1.66)
	Adj. res. = −0.9		Adj. res. = 0.9		
F30–F39 Mood (affective) disorders	41	52.6	158	56.8	OR = 1.08 (0.60–1.95)
	Adj. res. = −0.7		Adj. res. = 0.7		
Other diagnosis	21	26.9	75	27	(Reference)
	Adj. res. = 0		Adj. res. = 0		

**Table 3:** Institutional factors of suicide attempters and suicide completers.

	Suicide Attempters (Reference)		Suicide Completers		
	N	%	N	%	Statistics
**Patient’s status**					*χ^2^*(1) = 3.65, *p* = 0.056 BF_10_ = 0.96
Inpatient	52	66.7	152	54.7	(Reference)
	Adj. res. = 1.9		Adj. res. = −1.9		
Outpatient	26	33.3	126	45.3	OR = 1.66 (0.98–2.81)
	Adj. res. = −1.9		Adj. res. = 1.9		
**Type of health care**					*χ^2^*(1) = 0.03, *p* = 0.865 BF_10_ = 0.14
Psychiatric institutions	58	75.3	212	76.3	(Reference)
	Adj. res. = −0.2		Adj. res. = 0.2		
Other institutions	19	24.7	66	23.7	OR = 0.95 (0.53–1.71)
	Adj. res. = 0.2		Adj. res. = −0.2		
**Type of treatment**					*χ^2^*(1) = 6.48, *p* = 0.039 BF_10_ = 0.75
Medication only	38	64.4	107	48.4	(Reference)
	Adj. res. = 2.2, *p* = 0.01		Adj. res. = −2.2, *p* = 0.01		
Medication and psychotherapy	14	23.7	91	41.2	OR = 2.31 (1.18–4.53)
	Adj. res. = −2.5, *p* = 0.006		Adj. res. = 2.5, *p* = 0.006		
ECT, medication, and psychotherapy	7	11.9	23	10.4	OR = 1.17 (0.46–2.94)
	Adj. res. = 0.3		Adj. res. = −0.3		
**Type of medical error**					*χ^2^*(3) =16.06, *p* = 0.001 BF_10_ = 21.76
Insufficient level of observation and lack of safety measures	26	33.3	65	23.4	(Reference)
	Adj. res. = 1.8, *p* = 0.03		Adj. res. = −1.8, *p* = 0.03		
Inadequate suicide risk assessment	9	11.5	92	33.1	OR = 4.09 (1.80–9.30)
	Adj. res.= −3.7, *p* = 0.0001		Adj. res. = 3.7, *p* = 0.0001		
Inadequate/Delayed clinical/diagnostic/assessment	21	26.9	60	21.6	OR = 1.14 (0.58–2.24)
	Adj. res. = 1		Adj. res. = −1		
Inadequate treatment	22	28.2	61	21.9	OR = 1.11 (0.57–2.16)
	Adj. res. = 1.2		Adj. res. = −1.2		

## Data Availability

The data are not publicly available due to privacy restrictions, but may be obtained from the authors on reasonable request.
